# A Naturalistic Dynamic Monkey Head Avatar Elicits Species-Typical Reactions and Overcomes the Uncanny Valley

**DOI:** 10.1523/ENEURO.0524-19.2020

**Published:** 2020-07-07

**Authors:** Ramona Siebert, Nick Taubert, Silvia Spadacenta, Peter W. Dicke, Martin A. Giese, Peter Thier

**Affiliations:** 1 Department of Cognitive Neurology, Hertie Institute for Clinical Brain Research, University of Tübingen, Tübingen 72076, Germany; 2 Section for Computational Sensomotorics, Department of Cognitive Neurology, Hertie Institute for Clinical Brain Research and Werner Reichardt Centre for Integrative Neuroscience, University of Tübingen, Tübingen 72076, Germany; 3 Graduate School of Neural and Behavioural Sciences, International Max Planck Research School for Cognitive and Systems Neuroscience, University of Tübingen, Tübingen 72074, Germany

**Keywords:** dynamic expressions, naturalistic avatar, social cognition, uncanny valley

## Abstract

Research on social perception in monkeys may benefit from standardized, controllable, and ethologically valid renditions of conspecifics offered by monkey avatars. However, previous work has cautioned that monkeys, like humans, show an adverse reaction toward realistic synthetic stimuli, known as the “uncanny valley” effect. We developed an improved naturalistic rhesus monkey face avatar capable of producing facial expressions (fear grin, lip smack and threat), animated by motion capture data of real monkeys. For validation, we additionally created decreasingly naturalistic avatar variants. Eight rhesus macaques were tested on the various videos and avoided looking at less naturalistic avatar variants, but not at the most naturalistic or the most unnaturalistic avatar, indicating an uncanny valley effect for the less naturalistic avatar versions. The avoidance was deepened by motion and accompanied by physiological arousal. Only the most naturalistic avatar evoked facial expressions comparable to those toward the real monkey videos. Hence, our findings demonstrate that the uncanny valley reaction in monkeys can be overcome by a highly naturalistic avatar.

## Significance Statement

We introduce a new, naturalistic monkey avatar and validate it as an appropriate stimulus for studying primate social cognition by demonstrating that it elicits natural looking patterns and facial reactions in macaque monkeys rather than evoking an “uncanny” avoidance reaction. The fact that a degraded version of the avatar is able to evoke an uncanniness reaction confirms its existence in monkeys, supporting an evolutionary old behavioral commonality shared by monkeys and man. However, as this reaction can be overcome by a very naturalistic avatar, the uncanny valley is clearly not an inevitable consequence of high degrees of realism.

## Introduction

Faces and facial expressions provide crucial social information for humans and for monkeys. Experimental work investigating the neuronal underpinnings of social cognition and facial processing so far has been hampered by several challenges. First, the experimental subjects for invasive studies are usually monkeys, particularly rhesus macaques, while typical visual stimuli are images of humans (Leopold et al., 2006; Freiwald and Tsao, 2010; Chang and Tsao, 2017), disregarding considerable species differences. Second, the dynamic component of faces is often neglected, as many studies in monkeys have deployed static stimuli (Gothard et al., 2007; Hadj-Bouziane et al., 2008; Ramezanpour and Thier, 2020). Third, the stimuli largely lack standardization, compromising the collection of reliable data. Especially standardized videos of monkeys, e.g., producing certain facial expressions with specific gaze directions and all other variables constant are basically impossible to capture (Furl et al., 2012; Mosher et al., 2014; Shepherd and Freiwald, 2018). Fortunately, modern computer animation technology offers a solution: virtual, animated monkeys, i.e., monkey head avatars, providing full control over facial expression, eye and head movements. In our attempt to create a highly naturalistic monkey avatar, we built on a computer graphics (CG) model of a monkey head based on MRI scans, furnished with a physically naturalistic model of skin and fur, and controlled by ribbon-like muscle structures linked to motion capture-driven control points.

The usage of such stimuli, however naturalistic they may appear to us humans, requires stimulus validation: we cannot simply extrapolate from our human perception to the perception of an animal, whose differing anatomy, physiology, and cognitive capacities might create a different percept (Chouinard-Thuly et al., 2017). Catarrhine and human vision share many low-level characteristics (Weinstein and Grether, 1940; Shumake et al., 1968; De Valois et al., 1974), but this does not guarantee similar cognitive apprehension of the stimuli. How is the avatar experienced by monkeys? Do they find it strange, maybe even frightening? This is especially relevant in the light of previous work showing that macaque monkeys are susceptible to the “uncanny valley” phenomenon (Steckenfinger and Ghazanfar, 2009). This hypothesis by roboticist Masahiro Mori states that human affinity for robots directly increases with the degree of human-likeness, however, only up to a certain level. Beyond it, i.e., for very lifelike synthetic agents, the likeability drops suddenly into a deep uncanny valley, before rising again for real humans (Mori, 1970/2012). Anecdotal support for this hypothesis from computer games and animated movies (Butler and Joschko, 2009; Kaba, 2013; Kätsyri et al., 2017) has led many roboticists and graphic designers to deliberately aim for a “safe” non-human appearance to ensure avoiding the uncanny valley (Fong et al., 2003; Fabri et al., 2004).

Careful evaluation of our monkey avatar is especially important as it is animated (Chouinard-Thuly et al., 2017). Mori postulated that movement would deepen the uncanny valley and that unnaturalistic movement would even cause it (Mori, 1970/2012). Experimental studies show that the acceptability of a CG character (Piwek et al., 2014) and the recognition of a facial emotion (Tinwell et al., 2011) depend on the animation quality. These findings emphasize the importance of providing accurate, naturalistic facial animations for CG avatars, which is why we sought to avoid this pitfall by resorting to natural motion-capture driven facial animation.

The aim of this study was to test whether the uncanny valley reaction of monkeys can be overcome by an avatar with highly naturalistic motion and appearance and if the avatar’s facial expressions elicit natural reactions. To this end, we generated monkey face videos of incrementally naturalistic render types: unnaturalistic wireframe avatar, grayscale avatar, furless avatar, naturalistic avatar, and real monkey face. The faces displayed different expressions: neutral, fear grin, lip smack, threat and an artificial “blowing” expression to control whether the monkeys’ reactions are influenced by facial motion per se or by the emotional meaning of the facial expression. Two different video types (dynamic and static) of each render type/expression combination were produced. We showed all videos to eight rhesus macaques using the time spent looking at a stimulus as a measure of preference, widely practiced for nonverbal subjects such as monkeys (Gothard et al., 2004; Steckenfinger and Ghazanfar, 2009) or infants (Lewkowicz and Ghazanfar, 2012; Tafreshi et al., 2014). As the uncanny valley in humans is characterized by negative emotional valence (Mori, 1970/2012; Wang et al., 2015), we analyzed the monkeys’ physiological reactions (heart rate and pupil response) and their reactive facial expressions to elucidate whether they too might experience aversion.

## Materials and Methods

### Subjects

Data were collected from eight male rhesus macaques (*Macaca mulatta*; ages 7–16 years), born in captivity and pair-housed. All monkeys had previously been implanted with individually adapted titanium head posts to allow head immobilization in unrelated neurophysiological experiments and they had been trained to climb into a primate chair and to accept head fixation. All surgical procedures were conducted under aseptic conditions and full anesthesia (introduced with ketamine and maintained by inhalation of isoflurane and nitrous oxide, supplemented by intravenous remifentanil) with control of vital parameters (body temperature, CO_2_, O_2_, blood pressure, electrocardiogram). After surgery, monkeys were supplied with analgesics until full recovery. All animal procedures were approved by the local animal care committee (Regierungspräsidium Tübingen, Abteilung Tierschutz) and fully complied with German law and the National Institutes of Health’s *Guide for the Care and Use of Laboratory Animals*.

### Monkey head avatar

The basis of the avatar was a CG model of a monkey head based on MRI scans (3T head scanner, Siemens Magnetom Prisma). The surface mesh model derived from the scan was regularized, resulting in a mesh with 1.834 million polygons, which then was linked to a set of embedded ribbons that were modeled after the muscle anatomy of the macaque face (Parr et al., 2010). These elastic ribbons were linked to 43 control points, which correspond to the motion-captured markers, and which control the deformation of the mesh. The textures and skin material for the model were first painted by hand based on photograph reference and additional texture maps were derived to mimic all relevant layers of the skin using Adobe Photoshop. The monkey’s fur was created using Autodesk Maya’s XGen Interactive Grooming feature (https://knowledge.autodesk.com/support/maya/downloads/caas/CloudHelp/cloudhelp/2018/ENU/Maya-CharEffEnvBuild/files/GUID-496603B0-F929-45CD-B607-1CFCD3283DBE-htm.html), controlling the appearance and behavior of the simulated hair in terms of density, length, and direction maps. In order to generate the less naturalistic avatars, the highly naturalistic model was simplified in the following ways: (1) instead of modeling the fur structure in detail the face was modeled by a smooth surface with the same average color (furless); (2) the color information was discarded, modeling the face by a gray shaded smooth surface (grayscale); and (3) also the details of the surface structure were discarded, by subsampling the mesh with 30,940 polygons and connecting their points by smooth curves that follow the surface of the face, resulting in a wireframe picture with gray lines on a white background (wireframe). All movie frames were generated from the monkey head model using the Autodesk Arnold Renderer software.

### Dynamic expression modeling

**Figure 1.**
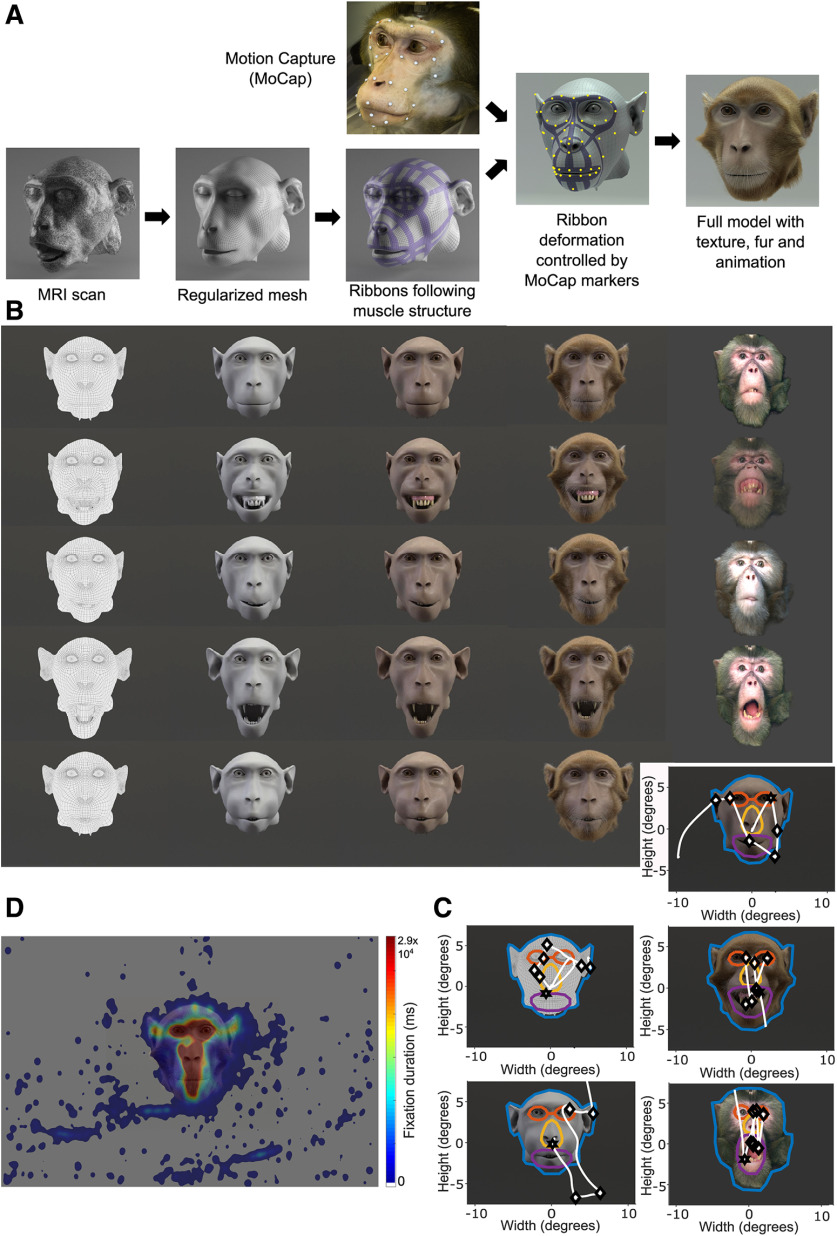
***A***, Schematic of the avatar generation process. ***B***, Overview of stimulus categories. Render types, columns left to right, Wireframe, grayscale, furless, naturalistic avatar, real monkey. Expressions, rows top to bottom, Neutral, fear grin, lip smacking, threat, artificial blowing. Images depict extreme frames of the expressions, which were also used for the static videos. ***C***, Example outlines of ROIs, within which fixations were analyzed, with overlaid example scanpaths on a wireframe avatar, neutral face (top left), grayscale avatar, blowing face (bottom left), furless avatar, lip-smacking face (top right), naturalistic avatar, fear grinning face (middle right), and real, threatening face (bottom right). Blue, Entire face ROI. Orange, Eyes ROI. Yellow, Nose ROI. Purple, Mouth ROI. White, Scanpath. Black star, First fixation. Black diamonds, Subsequent fixations. ROIs were manually drawn using the MATLAB *impoly* function and the coordinates of the ROI polygon were subsequently extracted using the *getPosition* function. ***D***, Heatmap of all fixations of all monkeys during the experiment. Heatmaps were created by plotting all fixations and convolving the image first in *x*, then in *y* direction with a Gaussian function of standard deviation 10 pixels, taking the duration of each fixation interval as the amplitude.

The facial movement of the avatar was based on motion capture data of real monkeys producing facial expressions. The monkeys were sitting in a primate chair with their head restrained. In order to attach the infrared reflecting tracer points to the skin, the monkeys’ face had to be shaved. The movement was recorded with a Vicon 1.8 Motion Capture System. In order to evoke facial expressions, interactions were initiated with the motion-captured monkey: (1) presenting a mirror for “lip smacking”; (2) showing a tool to elicit “fear grin”; and (3) staring at the monkey in a prolonged manner to elicit “threat.” The motion capture data were first preprocessed using Vicon NEXUS software to fill in missing marker trajectories and then segmented, selecting subsequences containing clear facial expressions (fear grin, lip smacking, open mouth threat, and neutral expressions). Motion capture data were recorded from two monkeys. The facial expressions used in this experiment were each based on one distinct expression by only one monkey. The resulting set of facial movements was time-normalized and further smoothed by approximating it using a Bayesian nonlinear dimension reduction method that combines Gaussian process latent variable models and Gaussian process dynamical models (Taubert et al., 2013). This algorithm also is suitable for online morphing between the dynamic expressions, a feature that was not used for the experiments in this paper, but for ongoing electrophysiological studies. Control experiments in humans verify that the algorithm outputs highly naturalistic facial motion (N. Taubert, M. Stettler, R. Siebert, S. Spadacenta, L. Sting, P.W. Dicke, P. Thier, M.A. Giese, unpublished observations). Figure 1*A* provides a schematic overview of the main steps of the avatar generation process.

### Visual stimuli

The visual stimuli consisted of 2-s-long video clips featuring the face of a monkey displaying either a dynamic or a static facial expression, the latter corresponding to the extreme frame of the dynamic videos. The portrayed monkey was either a computer-generated avatar or a filmed real monkey (monkey Ja). The real monkey video was recorded with a Canon Legria HF S30 Camcorder (8.6 megapixels, 25 frames/s) while the monkey was seated in a primate chair with his head immobilized in front of a uniform green background. The background was later removed using Adobe After Effects (https://www.adobe.com/products/aftereffects.html) showing only the head on a gray background to match avatar videos. Four render types of avatars with varying degree of realism were used (for details of generation, see above, Monkey head avatar): a very unnaturally appearing wireframe face; a textured, but grayscale, still quite unnaturalistic avatar; a slightly more naturalistic, colored, but still furless avatar; the highly naturalistic monkey head avatar including fur and facial details such as wrinkles. All these monkey faces of different render type displayed one of four distinct species-specific facial expressions, or (in case of the avatars) an additional artificial expression, whereby the depicted monkey was blowing up its cheeks, a behavior never shown by real rhesus monkeys. The species-specific expressions consisted of fear-grin (a fearful or submissive reaction), lip smacking (an affiliative, peaceful gesture), and open mouth threat (an intimidating, aggressive display). This yielded 48 different videos. See Figure 1*B* for an overview of the stimulus matrix of the 24 static views.

### Setup and paradigm

The monkey subjects sat in a primate chair with their head restrained inside a booth at a distance of 60 cm in front of a 24-inch monitor (1920 × 1080 screen resolution, 144-Hz refresh rate). Each trial started with the presentation of a central fixation dot (2° diameter) for 1 s to draw the monkeys’ attention to the screen, followed by 2-s videos, with a 1-s intertrial interval. The stimuli were presented via the NREC open source control system (https://nrec.neurologie.uni-tuebingen.de). The monkeys’ eye position and pupil size (area) were monitored with an eye tracker (EyeLink 1000, sampling rate 1000 Hz). They could freely move their eyes and were rewarded with a drop of water after each trial, as long as they kept their gaze direction within the boundaries of the monitor (fixation window 47° by 28° visual angle). The videos spanned 22° horizontally and 15° vertically. We conducted two different experiments. In experiment 1, a single video was presented centrally in the middle of the monitor (= 48 different trials), and in experiment 2, two videos of the same facial expression and motion type (dynamic or static), but of different render type were presented side by side, centered at −11° and 11° horizontally from the middle of the screen, respectively (= 184 different trials). Videos were played at 60 frames/s on a gray background, and trials were presented in pseudo-random order. The electrocardiogram was measured as the electrical potential difference between an electrode attached to the monkeys’ head post and a second electrode attached to the metal grid on the bottom of the primate chair on which the monkeys were sitting and was recorded using the Open Ephys recording system (http://www.open-ephys.org/, sampling rate 5000 Hz). Finally, the monkeys’ own reactive facial expressions to the videos were filmed with a Canon Legria HF S30 Camcorder (8.6 megapixels, 25 frames/s). Each monkey completed between six and 11 sessions (*n* = 62 sessions total).

### Data analysis

All data were analyzed using MATLAB R2018a (MathWorks).

#### Looking behavior

Eye movement data were smoothed and eye velocity was determined by calculating the first derivative of the eye movement signal using a second order Savitzky–Golay filter of window size 10. Fixations were defined as time periods of at least 100 ms duration during which eye velocity did not exceed 20°/s. The mean coordinates during a particular fixation period served as the eye position during this fixation and the duration of each fixation was calculated. Regions of interest (ROIs) constituting the face (as opposed to the rest of the screen), the eyes, mouth, and nose were determined in all stimulus videos by manually outlining those areas closely along their borders on the frame with the maximum expression, using the same ROI for the static and dynamic video (Fig. 1*C*). For each trial, all eye fixations that fell within the respective ROI coordinates were identified. The fixations on each ROI were tallied up, yielding the number of fixations on the entire face and on the individual face parts. Fixations of the nose were only included in the feature index (see below) but not further analyzed individually due to their limited information content on emotional states. The first fixation was discarded when calculating the number of fixations on face parts as it usually fell close to the position of the fixation dot initiating the trial, in the vicinity of the nose. Additionally, the cumulative and mean fixation durations on the face and on face parts per trial were calculated, as well as the total looking time, which represents the total amount of time the eye position stayed on the face (or eyes/mouth) in one trial, including fixations as well as saccades. Two measures of exploration were computed: (1) the exploration distance within the face, which is the sum of the distances of all face fixation points from the geometrical fixation center of gravity, providing information about whether the monkeys scrutinize the entire face thoroughly or rather make several fixations on one or few (relevant or irrelevant) facial features; (2) a feature index, which is the difference between the number of fixations on relevant face parts (eyes, mouth, and nose) and the number of fixations on irrelevant face parts (remaining facial regions), divided by the total fixation number:

$$\left(fixation\;number_{rel}–fixation\;number_{irrel}\right)/fixation\;number_{total}.$$

The resulting values lie between −1 and +1, whereby negative values indicate more fixations on irrelevant compared with relevant areas, and positive values indicate relatively more fixations on relevant areas, allowing us to test whether all stimuli equally induce typical scanpaths emphasizing the eyes, mouth, and nose (Loughland et al., 2002; Delerue et al., 2010) rather than potential abnormalities in the skin or fur.

#### Pupil size

Pupil size is linked to arousal and has been shown to respond to the social relevance of stimuli in rhesus macaques (Ebitz et al., 2014). In order to gauge pupil size, first, noise was removed from the raw pupil size signal by deploying a second order Savitzky–Golay filter (window size 20 samples). Eye-blink artifacts were eliminated by detecting values smaller than −4 (arbitrary eye tracker area units), then discarding all samples up to 100 ms before and 100 ms after the signal dropped below the threshold of −4, and finally linearly interpolating the signal across the resulting gap. The pupil size signal was normalized for a given session by translating it into *z* scores. Subsequently, the signal was divided into bins of 250 ms and averaged per bin. The resulting averages were the basis of comparison between dynamic and static videos and between expressions. Comparison between render types was not possible because of luminance differences between the avatar versions.

#### Heart rate

Heart rate variability (HRV) is controlled by the sympathetic and parasympathetic nervous system, whereby high HRV at rest is an indicator of good health and increased HRV is associated with decreased arousal and, conversely, lowered HRV is associated with heightened arousal. Studies in humans have reported that it is possible to induce measurable changes in HRV by visual emotional stimulation (Choi et al., 2017) and also monkey cardiac physiology has been shown to be responsive to affective video content (Bliss-Moreau et al., 2013). Hence, the electrocardiogram was recorded continuously throughout each session. Heart data from monkeys C, K, and L could not be recorded due to technical problems. Afterwards, the signal was first bandpass filtered between 1 and 40 Hz using a rectangular window, then down-sampled to 1000 Hz and subsequently smoothed using a second order Savitzky–Golay filter (window size 20 samples). Artifacts, e.g., due to movements of the monkey, were cut out manually from the signal and trials containing artifacts were subsequently discarded. QRS-complexes were identified using the MATLAB *findpeaks* function and verified by visual inspection. Then, R-R intervals were calculated, and the heart rate in beats per minute (bpm), as well as the root mean square of successive differences (RMSSD) as a measure of HRV (Shaffer and Ginsberg, 2017) were computed for each trial:

$$RMSSD=\surd \left({\left(RR_{1}-RR_{2}\right)}^{\text{2}}+...+{\left(RR_{n-1}–RR_{n}\right)}^{\text{2}}\right).$$

#### Reactive facial expressions

Video recordings of the monkeys’ reactions were inspected visually. Only monkeys C, E, and P showed clear facial reactions toward the videos and were included in the analysis. Monkeys C and E reacted in the first block only, whereas monkey P reacted throughout the first four sessions (*n* = 6). Video recordings of the monkeys’ reactive facial expressions were scored manually, blind to the experimental condition, by judging whether the monkey lip smacked, fear grinned, behaved agitatedly (tension yawns, teeth grinding) or showed no reaction during the timeframe of each trial (other expressions, like open mouth threat, were never shown). Afterwards, the probability for each of the three monkeys to show each of these four types of reactions was calculated for all 48 different stimuli.

#### Statistical analysis

First, the within-subject mean was calculated for each condition and each variable. All subsequent comparisons were based on these means. As all dependent variables were not normally distributed as shown by Kolmogorov–Smirnoff tests for each variable (*p *<* *0.05), we could not use a parametric multi-factorial repeated-measures ANOVA. Instead, Friedman’s nonparametric ANOVAs for related samples were deployed to test for the effects of render type, expression and video type (dynamic vs static) on all parameters individually. Blowing avatars had to be excluded from the statistical analysis of render type effects because of the absence of a natural blowing expression in videos of real monkeys. Likewise, to assure an equal number of render types per expression category for statistical testing, blowing expressions were omitted from the expression effects analysis. In order to determine pairwise differences with the blowing expression, additional Friedman’s ANOVAs for expression effects were conducted omitting the real videos. First, the effects of render type, expression, and video type were examined within the entire dataset. Then, the data were divided into dynamic and static conditions and analyzed for the effects or render type and expression separately. Finally, the data were also split by expression to test for the render type effect within each expression individually, and split by render type to test for the expression effect within each render type individually. *Post hoc* pairwise multiple comparisons were performed using Dunn and Sidák’s approach. We chose a significance level of *p *<* *0.05 for all comparisons.

## Results

Three different outcomes were conceivable regarding the render type. (1) If the naturalistic avatar is perceived as a real monkey and the uncanny valley does not exist, all reactions toward the naturalistic avatar and the real monkey should be the same, and the reactions toward the other avatars should differ. (2) If the uncanny valley exists in the form predicted by Mori, monkeys should avoid looking at the synthetic face with highest realism, our naturalistic avatar. (3) If an uncanny valley exists, but high realism is not the critical factor eliciting it, instead eerie stimulus features in general, monkeys should avoid the strange-looking less naturalistic avatars.

Assuming that the uncanny valley in humans and monkeys are equivalent phenomena, the least preferred stimuli should elicit feelings of aversion, which in turn should induce physiological arousal and reactive facial expressions of fear or agitation, whereas we expected higher affinity for a character to evoke affiliative facial reactions, i.e., lip smacking.

With respect to the video type (dynamic vs static), the uncanny valley hypothesis makes two predictions: movement would deepen the aversion toward an uncanny stimulus, and unnaturalistic movement would cause an otherwise acceptable artificial character to descend into the uncanny valley. If this pertains, the static avatar at the bottom of the valley should be even more avoided when moving. If no valley is apparent in the static condition, but one emerges in the dynamic condition, this would mean that the animation of the avatar is flawed.

Regarding the facial expression category of the stimulus, monkeys should prefer the expressions with the highest ethological importance, i.e., threat (negative) and lip smacking (positive), especially in the dynamic condition, which should also induce physiological arousal. We expected the monkeys’ facial reaction to be predominantly governed by the viewed render type and less contingent on the viewed facial expression.

### Looking behavior (experiment 1)

The monkeys showed a general interest in looking at the faces of the centrally presented stimulus videos and largely ignored the surrounding background, as evident from Figure 1*D*. The primary target of fixation were the eyes with 18.85% of all fixations on the face, the mouth received 14.60%.

#### Influence of render type

**Figure 2.**
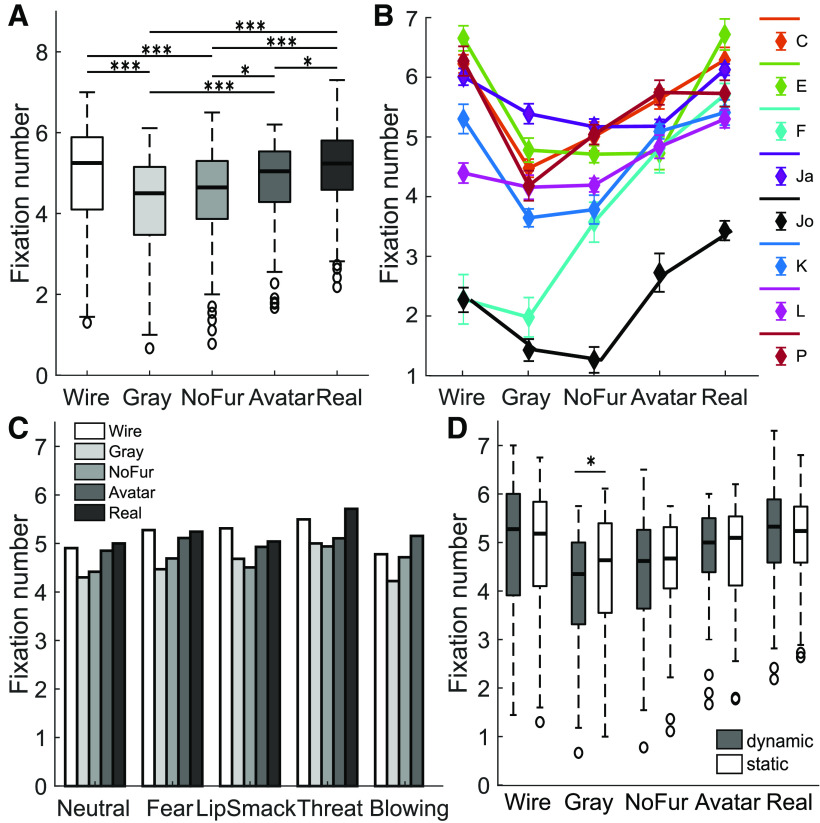
Number of fixations on different render types (*N* = 8). ***A***, Boxplots show fixation numbers per render type (render type effect size: χ^2^(4) = 72.60, *p *<* *0.001). ***B***, Fixation numbers for all monkeys individually. Markers indicate within-subject mean ± SEM. ***C***, Median fixation numbers separated by expression; effect was significant within each expression category except for blowing (marginally significant). Neutral: χ^2^(4) = 19.09, *p *<* *0.001; fear: χ^2^(4) = 17.80, *p *=* *0.0013; lip smack: χ^2^(4) = 19.39, *p *<* *0.001; threat: χ^2^(4) = 19.90, *p *<* *0.001; blowing: χ^2^(3) = 7.37, *p *=* *0.061. ***D***, Boxplots show fixation numbers on dynamic and static videos within each render type category. Significant differences between dynamic and static videos were found for grayscale avatars only. Wireframe: χ^2^(1) = 0.026, *p *=* *0.87; grayscale: χ^2^(1) = 5.77, *p *=* *0.016; furless: χ^2^(1) = 0.026, *p *=* *0.87; avatar: χ^2^(1) = 0.40, *p *=* *0.53; real: χ^2^(1) = 1.13, *p *=* *0.29; *p <* *0.05, **p <* *0.01, ***p <* *0.001.

The render type had a significant effect on all parameters used to characterize the looking behavior, except for the feature index (measure for focus on relevant vs irrelevant face parts, see Materials and Methods), in the entire dataset from eight monkeys comprising dynamic and static as well as all expression conditions. The monkeys looked most at the videos showing the real monkey and the unnaturalistic wireframe head, followed by the naturalistic avatar, whereas they avoided looking at the grayscale and furless avatars. The naturalistic avatar, wireframe avatar and real monkey were significantly preferred over the grayscale and furless avatars. This pattern was seen in the number of fixations (Fig. 2*A*) and the total looking time (although pairwise differences were not always significant for looking time), cumulative fixation duration results were less clear. Interestingly, the lower number and total duration of looks at the gray and furless faces were accompanied by a longer mean fixation duration and a greater focus on the eyes at the expense of the mouth and by a reduced exploration distance. Real and naturalistic avatar faces were explored the most. A differently weighted focus on relevant facial features compared with irrelevant areas as represented in the feature index was not observed. Compare Table 1 for an overview of all investigated parameters.

**Table 1:** Render type effect on all looking parameters investigated: cumulative fixation duration (FixDur), fixation number (FixNum), mean fixation duration (MeanFix), and total looking time (LookT) on the entire face, the eyes and the mouth, as well as exploration distance (ExplorDistance) and the feature index

Lookingparameter	Wireframe		Grayscale		Furless		Avatar		Real		χ^2^ (df = 4),*n* = 64, *p* value
	median (IQR)	Diff	median (IQR)	Diff	median (IQR)	Diff	median (IQR)	Diff	median (IQR)	Diff	
FixDur face	1372.38 (1004.95–1528.14)	–	1264.38 (909.91–1491.12)	R	1307.35 (972.22–1500.50)	–	1287.79 (1067.57–1497.17)	–	1291.11 (1107.00–1503.56)	G	14.34, 0.0063
FixNum face	5.25 (4.10–5.88)	G,F	4.50 (3.47–5.15)	W,A,R	4.65 (3.87–5.30)	W,A,R	5.05 (4.29–5.54)	G,F,R	5.24 (4.59–5.81)	G,F,A	72.60, 0.000
MeanFix face	257.90 (202.76–297.55)	G,F	281.20 (219.47–335.81)	W,R	279.79 (215.82–318.47)	W	266.05 (223.89–317.37)	–	255.79 (209.76–304.01)	G	14.31, 0.000
LookT face	1747.15 (1481.48–1904.80)	–	1645.17 (1332.39–1805.54)	R	1632.71 (1325.82–1818.56)	R	1659.63 (1464.91–1869.56)	–	1722.50 (1490.59–1859.79)	G,F	20.16, 0.000
FixDur eyes	131.69 (43.25–333.02)	–	154.24 (40.20–340.94)	R	208.69 (79.64–424.49)	R	148.73 (54.76–258.94)	–	120.68 (57.80–196.25)	G,F	22.76, 0.000
FixNum eyes	0.69 (0.23–1.45)	–	0.79 (0.14–1.60)	–	0.88 (0.41–1.63)	R	0.73 (0.19 –1.16)	–	0.59 (0.30–0.95)	F	13.96, 0.0074
MeanFix eyes	87.69 (34.21–160.47)	F	99.03 (27.18–185.81)	–	111.18 (41.15–200.42)	W,R	86.06 (46.71–137.05)	–	88.52 (41.03–113.22)	F	17.82, 0.0013
LookT eyes	206.27 (68.00–450.86)	–	233.94 (70.78–433.51)	R	264.91 (113.93–543.33)	R	225.89 (88.39–331.98)	–	150.89 (85.44–235.24)	G,F	22.95, 0.000
FixDur mouth	114.21 (44.21–348.00)	R	85.50 (22.00–238.72)	R	83.55 (36.43–255.68)	R	134.61 (37.64–322.05)	R	295.09 (154.16–502.11)	W,G,F,A	31.41, 0.000
FixNum mouth	0.47 (0.20–1.10)	R	0.30 (0.13–0.76)	R	0.35 (0.15–0.88)	R	0.60 (0.18–1.00)	R	1.05 (0.65–1.75)	W,G,F,A	49.13, 0.000
MeanFix mouth	88.65 (38.25–170.35)	R	66.22 (21.51–134.25)	R	72.56 (21.61–167.42)	R	85.35 (34.71–180.25)	R	184.02 (121.85–234.05)	W,G,F,A	32.15, 0.000
LookT mouth	185.02 (89.82–508.29)	R	140.38 (48.50–486.70)	R	190.06 (70.80–458.94)	R	216.90 (76.28–545.16)	R	663.86 (357.08–890.90)	W,G,F,A	79.75, 0.000
ExplorDistance	10.38 (6.17–13.22)	F	10.57 (5.25–12.73)	A,R	10.91 (6.98–12.67)	W,A,R	12.57 (7.26–14.25)	G,F	11.82 (7.52–14.12)	G,F	50.91, 0.000
Feature index	–0.13 (–0.45–0.064)	–	–0.23 (–0.48–0.19)	–	–0.27 (–0.42–0.15)	–	–0.29 (–0.47–0.070)	–	–0.32 (–0.49– 0.00)	–	7.16, 0.13

Table shows group medians and interquartile range (IQR) of all eight subjects per condition, results of Friedman’s ANOVA (last column) with test statistic value (χ^2^), degrees of freedom (df), number of values per condition included in the statistical analysis (*n*), and significance level (*p* value) and shows from which groups the respective group differed significantly (Diff, *p *<* *0.05) according to Dunn and Sidák’s *post hoc* multiple comparisons approach (W = wireframe, G = grayscale, F = furless, A = avatar, R = real). To be able to apply Friedman’s ANOVA, trials with a blowing avatar were excluded from the analysis to assure the same number of expressions per render type category (no blowing expression in the real video). Effects were regarded as statistically significant at *p *<* *0.05; *p* values <0.001 were rounded to 0.000.

Analyzing reactions to dynamic and static stimuli separately yielded the same pattern of effects of render type as the analysis of the pooled data (fixation number dynamic: χ^2^(4) = 51.11, *p *<* *0.001; static: χ^2^(4) = 24.23, *p *<* *0.001). As evident in Figure 2*B*, there were interindividual differences in the monkeys’ performance and preferences, but a clear avoidance of the gray and furless avatars was shown by six out of eight monkeys. The render type effect on fixation number was consistent within all expressions individually; however, within the artificial blowing expression, it was only marginally significant (Fig. 2*C*).

We would argue that fixation number, the parameter that exhibited the most robust effect of render type, is indeed the most appropriate measure of preference as it is not influenced by the limitations that compromise the informative value of the others: cumulative fixation duration can only change markedly in monkeys that generally look less at the face and more on the surrounding screen. For monkeys who have a high baseline for looking at the face, a further increase in fixation number must necessarily lead to a decrease in total fixation time as the saccades between subsequent fixations also require time and the duration of the trial is fixed, which is what we observed in our data. Total looking time, comprising fixations and saccades on the face, avoids this problem but introduces another, because it includes times during which no processing of the visual input takes place, i.e., during re-fixation saccades. Like cumulative fixation duration, mean fixation duration suffers from the fact that every video has the same duration, which monkeys most likely realize very quickly. Hence, a monkey wishing to scrutinize a given video more intensively must necessarily decrease the duration of an individual fixation. Although mean fixation duration might not carry much information about preference, it can still reflect the saliency or importance of the respective fixation target (e.g., eyes or mouth).

#### Influence of expression

**Figure 3.**
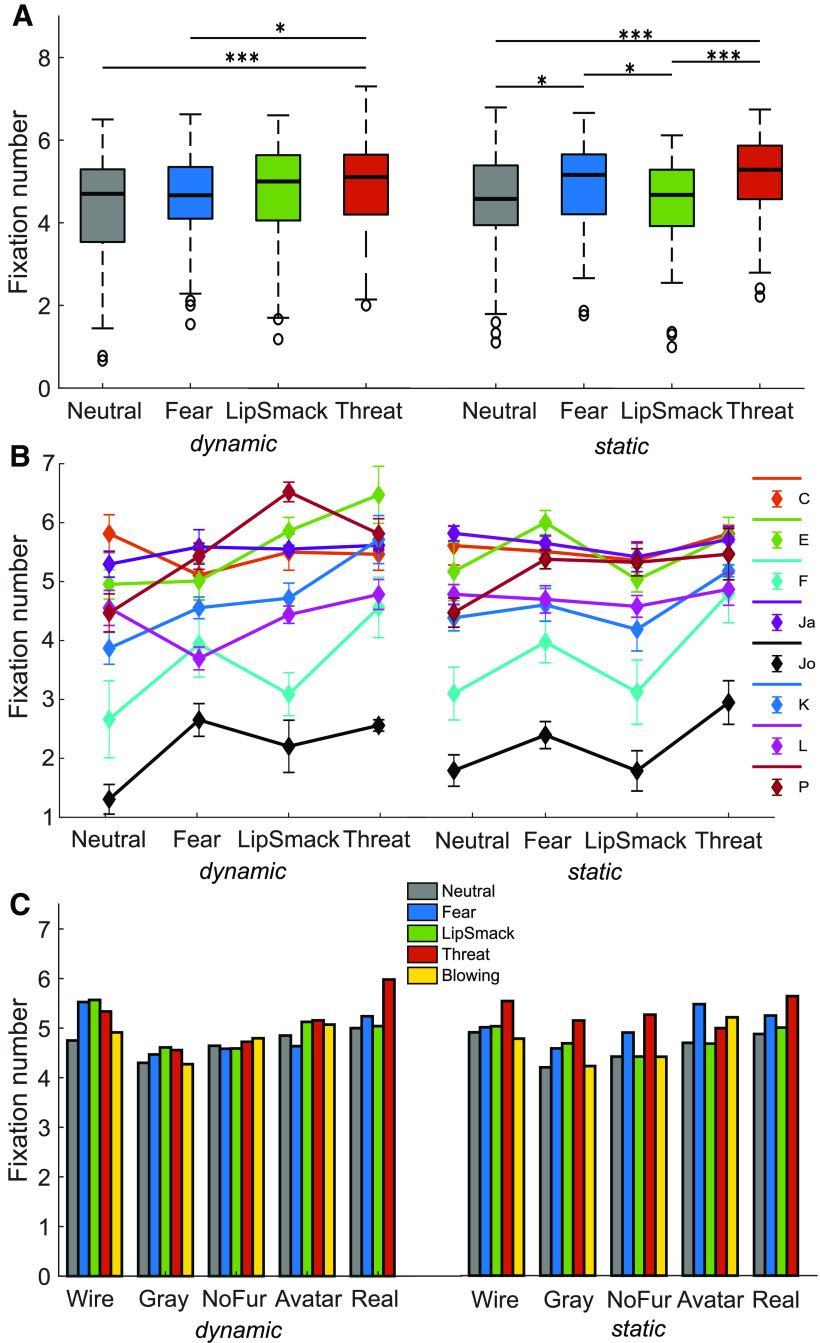
Number of fixations on different expressions (*N* = 8). ***A***, Boxplots show fixation numbers per dynamic expressions (left), expression effect size: χ^2^(3) = 19.27, *p *<* *0.001; and static expressions (right), expression effect size: χ^2^(3) = 25.82, *p *<* *0.001. ***B***, Fixation numbers for all monkeys individually, dynamic expressions (left) and static expressions (right). Markers indicate within-subject mean ± SEM. ***C***, Median fixation numbers separated by render type. Left, Dynamic expressions: wireframe: χ^2^(4) = 9.02, *p *=* *0.061; gray: χ^2^(4) = 8.51, *p *=* *0.075; no fur: χ^2^(4) = 7.16, *p *=* *0.13; avatar: χ^2^(4) = 13.01, *p *=* *0.56; real: χ^2^(3) = 2.54, *p *=* *0.47. Right, Static expressions, effect was significant within the no fur category and marginally significant within the gray category: wireframe: χ^2^(4) = 6.80, *p *=* *0.15; gray: χ^2^(4) = 9.48, *p *=* *0.050; no fur: χ^2^(4) = 11.56, *p *=* *0.021; avatar: χ^2^(4) = 4.38, *p *=* *0.36; real: χ^2^(3) = 2.88, *p *=* *0.41; *p <* *0.05, **p <* *0.01, ***p <* *0.001.

The type of facial expression shown in the videos influenced the looking behavior differently depending on whether the video was static or dynamic as documented by Figure 3*A*. Table 2 summarizes the results of all parameters within dynamic, static and pooled data. For static faces, comprising all render types, the monkeys looked most at the threatening expression, followed by fear grin, and significantly less at the lip smacking and neutral faces. When the video content was dynamic, the most fixations were counted on threatening displays, this time followed by lip-smacking faces, whereby fear grinning and neutral faces were looked at least. The artificial expression with the blown-up cheeks received an intermediate amount of looks both when static or moving. This was measured in fixation number as well as total looking time, less clearly in cumulative fixation duration. Mean fixation duration was not distinctly modulated. The patterns were more variable between monkeys than the render type effect (Fig. 3*B*), but appeared rather consistent over render types (Fig. 3*C*). However, the expression effect did not reach significance for individual categories other than furless static faces (grayscale static marginally significant), most probably due to lack of statistical power resulting from the small sample size when the data were split up both by render type and video type.

Attention to the eyes did not differ between expressions, but the mouth was looked at most for fear grins, followed by open mouth threats, then lip smacks and least for neutral and blowing expressions, which was observed both for both dynamic and static expressions, probably a reflection of feature saliency. The amount of exploration was significantly increased for threatening displays. The feature index reflected the aforementioned effects of looks on the mouth, in that it was greatest for fear and threat, then lip smacking, and smallest for neutral and blowing faces (Table 2).

#### Influence of video type

When examining the effect of movement within a face throughout the entire dataset comprising all render types and expressions, presenting dynamic faces rather than static ones did not lead to overall more, but to on average longer fixations. Movement caused a slightly stronger focus on the mouth, the part of the faces exhibiting the most pronounced movements, whereas static faces drew more attention to the eyes. This is shown by an increased mean fixation duration and marginally increased the cumulative fixation duration on dynamic faces compared with static ones. The eyes of static faces received a higher fixation number, cumulative fixation duration and total looking time, whereas the mean fixation duration on the mouth was longer for dynamic videos (for an overview of all parameters, see Table 3). When looking at the video type effect within each expression separately, fixation number did not differ significantly, but it was revealed that movement particularly increased the mean fixation duration on fear grinning (χ^2^(1) = 4.90, *p *=* *0.027), threatening (χ^2^(1) = 19.60, *p *<* *0.001), and blowing (χ^2^(1) = 4.50, *p *=* *0.034) faces, whereas it increased the total looking time at lip smacking displays (χ^2^(1) = 8.10, *p *=* *0.0044). Dynamic videos especially drew away attention from the eyes of lip-smacking faces, leading to shorter fixations (mean fixation duration: χ^2^(1) = 5.16, *p *=* *0.023), whereby the gaze was instead fixated longer on the mouth (mean fixation duration: χ^2^(1) = 10.53, *p *=* *0.0012). Also, fixations were deflected faster from the eyes of blowing faces (mean fixation duration: χ^2^(1) = 7.26, *p *=* *0.0071), possibly in favor of the salient movement at the cheeks. Separate analysis within each render type showed that dynamic content prolonged the mean fixation duration especially on the naturalistic avatar (χ^2^(1) = 12.10, *p *<* *0.001) and on the grayscale avatar (χ^2^(1) = 10.00, *p *=* *0.0016). Notably, the number of fixations on moving faces was less for grayscale avatars only (χ^2^(1) = 5.77, *p *=* *0.016; Fig. 2*D*), with a decreased looking time (χ^2^(1) = 6.40, *p *=* *0.011) and decreased mean fixation duration (χ^2^(1) = 5.16, *p *=* *0.023) on the eyes.

**Table 2:** Expression effect for all data and for dynamic and static conditions separately on all looking parameters investigated: Cumulative fixation duration (FixDur), fixation number (FixNum), mean fixation duration (MeanFix), total looking time (LookT) on the entire face, the eyes and the mouth, as well as exploration distance (ExplorDistance) and the feature index

Lookingparameter	Neutral		Fear		Lip smack		Threat		Blowing		χ^2^ (df = 3), *n* = 80 (all)/*n* = 40 (dyn./ stat.), *p* value
	median (IQR)	Diff	median (IQR)	Diff	median (IQR)	Diff	median (IQR)	Diff	median (IQR)	Diff	
FixDur face	1248.40 (945.96–1504.73)	T	1285.90 (1007.32–1509.05)	-	1274.78 (1036.78–1472.54)	-	1359.36 (1116.01–1521.50)	N	1295.06 (906.30–1512.56)	-	13.19, 0.0043
Dynamic	1252.29 (1024.79–1499.55)	T	1261.65 (1023.37–1481.34)	T	1334.24 (1089.78–1495.33)	-	1413.88 (1187.31–1557.18)	N,F	1368.56 (866.48–1495.15)	-	11.61, 0.0088
Static	1231.20 (895.70–1512.90)	-	1359.37 (1007.33–1515.76)	-	1219.72 (964.70–1470.73)	-	1300.02 (1045.58–1474.63)	-	1248.46 (946.79–1531.31)	-	9.81, 0.020
FixNum face	4.60 (3.74–5.34)	F,T	4.78 (4.17–5.59)	N,T	4.75 (4.00–5.50)	T	5.16 (4.39–5.75)	N,F,L,B	4.75 (3.79–5.33)	T	36.13, 0.000
Dynamic	4.70 (3.54–5.29)	T	4.67 (4.10–5.35)	T	5.00 (4.06–5.64)	-	5.11 (4.20–5.65)	N,F	4.79 (3.61–5.32)	-	19.27, 0.000
Static	4.59 (3.95–5.40)	F,T	5.17 (4.21–5.66)	N,L	4.68 (3.93–5.29)	F,T	5.29 (4.58–5.88)	N,L	4.75 (3.79–5.38)	-	25.82, 0.000
MeanFix face	272.72 (215.33–327.00)	-	268.26 (221.70–314.29)	-	271.51 (214.84–326.07)	-	262.75 (214.94–314.11)	-	272.27 (228.53–329.26)	-	1.67, 0.64
Dynamic	276.33 (222.06–359.12)	-	270.66 (224.89–318.06)	-	262.50 (217.53–316.61)	-	277.96 (228.70–337.22)	-	275.36 (230.03–337.23)	-	3.57, 0.31
Static	264.10 (201.13–321.86)	-	266.48 (203.82–301.40)	-	273.47 (208.47–342.47)	-	250.87 (205.81–290.07	-	263.72 (218.54–312.33)	-	8.04, 0.045
LookT face	1583.35 (1324.88–1822.86)	F,T	1676.00 (1482.22–1859.79)	N,B	1651.82 (1414.69–1816.48)	T	1741.56 (1515.20–1897.24)	N,L,B	1659.39 (1315.08–1804.15)	F,T	21.98, 0.000
Dynamic	1583.35 (1356.10–1824.22)	T	1655.18 (1487.83–1847.60)	T	1709.45 (1490.44–1860.99)	-	1769.91 (1559.55–1913.73)	N,F,B	1691.35 (1310.83–1796.10)	T	17.55, 0.000
Static	1579.56 (1224.25–1814.67)	F	1723.87 (1473.90–1882.95)	N,L,B	1605.48 (1343.86–1791.33)	F	1720.75 (1484.35–1849.24)	-	1614.57 (1355.80–1841.81)	F	12.99, 0.0047
FixDur eyes	132.45 (47.45–309.08)	-	137.44 (52.25–295.85)	-	148.83 (58.52–277.27)	-	157.44 (49.95–295.45)	-	161.70 (36.61–337.46)	-	0.61, 0.89
Dynamic	132.45 (40.79–269.58)	-	171.50 (52.25–267.40)	-	141.52 (58.52–254.95)	-	163.75 (64.73–280.55)	-	86.83 (20.15–282.23)	-	4.92, 0.18
Static	146.00 (53.75–316.96)	-	108.55 (49.13–315.10)	-	174.06 (58.98–355.03)	-	152.92 (43.42–297.25)	-	186.05 (85.84–346.96)	-	4.24, 0.24
FixNum eyes	0.68 (0.21–1.25)	-	0.61 (0.20–1.27)	-	0.75 (0.32–1.38)	-	0.75 (0.27–1.41)	-	0.65 (0.15–1.40)	-	0.16, 0.98
Dynamic	0.60 (0.14–1.13)	-	0.72 (0.31–1.24)	-	0.65 (0.33–1.14)	-	0.71 (0.34–1.40)	-	0.44 (0.11–1.25)	-	3.21, 0.36
Static	0.80 (0.30–1.38)	-	0.56 (0.17–1.31)	-	0.76 (0.30–1.50)	-	0.84 (0.24–1.46)	-	0.79 (0.24–1.56)	-	4.48, 0.21
MeanFix eyes	84.13 (37.22–155.71)	-	91.27 (42.00–158.89)	-	92.07 (36.78–166.40)	-	104.53 (37.88–153.84)	-	97.34 (33.61–173.51)	-	0.090, 0.99
Dynamic	78.45 (37.22–149.58)	-	93.22 (44.70–152.67)	-	89.26 (31.24–161.60)	-	108.00 (34.13–170.79)	-	54.27 (20.15–158.55)	-	5.93, 0.12
Static	97.98 (37.33–159.86)	-	74.61 (38.64–158.89)	-	99.60 (49.19–182.44)	-	85.94 (42.83–136.75)	-	100.56 (46.76–176.23)	-	6.73, 0.081
LookT eyes	207.33 (78.79–394.69)	-	190.17 (79.54–391.33)	-	207.94 (85.69–406.61)	-	228.67 (73.89–359.25)	-	200.88 (81.15–446.29)	-	1.18, 0.76
Dynamic	198.74 (72.49–351.97)	-	204.37 (79.54–371.99)	-	190.75 (75.01–342.14)	-	236.72 (86.73–359.25)	-	162.28 (42.97–412.26)	-	2.31, 0.51
Static	229.50 (93.14–441.49)	-	174.60 (85.16–423.28)	-	221.06 (115.55–483.18)	-	218.98 (73.89–359.33)	-	236.95 (108.33–456.71)	-	7.39, 0.060
FixDur mouth	42.77 (0.00–105.28)	F,L,T	332.88 (164.00–492.01)	N,L,B	97.60 (32.34–267.93)	N,F,T	194.50 (86.36–435.15)	N,L,B	40.36 (0.00–123.09)	F,T	138.48, 0.000
Dynamic	53.08 (0.00–101.61)	F,L,T	318.10 (175.22–425.38)	N,L,B	169.83 (37.80–308.61)	N,F,T	215.08 (63.03–431.31)	N,L,B	46.55 (0.00–164.79)	F,T	60.61, 0.000
Static	35.21 (0.00–105.60)	F,T	364.00 (162.89–516.43)	N,L,B	68.04 (19.36–147.10)	F,T	191.39 (105.68–444.44)	N,L,B	32.61 (0.00–75.89)	F,T	82.44, 0.000
FixNum mouth	0.18 (0.00–0.35)	F,L,T	1.10 (0.71–1.45)	N,L,B	0.33 (0.13–0.80)	N,F,T	0.76 (0.38–1.61)	N,L,B	0.13 (0.00–0.44)	F,T	154.06, 0.000
Dynamic	0.18 (0.00–0.30)	F,L,T	1.15 (0.68–1.40)	N,L,B	0.52 (0.19–0.86)	N,F,T	0.73 (0.33–1.50)	N,L,B	0.13 (0.00–0.53)	F,T	68.47, 0.000
Static	0.17 (0.00–0.42)	F,T	1.05 (0.75–1.69)	N,L,B	0.25 (0.13–0.69)	F,T	0.83 (0.50–1.69)	N,L,B	0.13 (0.00–0.28)	F T	91.15, 0.000
MeanFix mouth	32.29 (0.00–76.13)	F,L,T	185.52 (99.68–250.33)	N,L,B	81.24 (22.28–147.90)	N,F,T	124.94 (68.72–212.58)	N,L,B	37.44 (0.00–91.23)	F,T	110.95, 0.000
Dynamic	31.64 (0.00–83.43)	F,L,T	185.96 (115.80–260.35)	N,L,B	108.88 (37.80–193.73)	N,F	141.08 (54.68–231.49)	N,B	46.55 (0.00–109.25)	F,T	52.05, 0.000
Static	32.71 (0.00–76.13)	F,T	183.11 (82.40–242.64)	N,L,B	44.37 (19.36–106.97)	F,T	105.57 (78.54–198.50)	N,L,B	29.39 (0.00–66.74)	F,T	61.35, 0.000
LookT mouth	95.07 (31.49–217.05)	F,L,T	669.77 (389.74–839.84)	N,L,T,B	167.30 (59.77–465.01)	N,F,T	296.59 (144.95–679.65)	N,F,L,B	75.64 (24.02–211.48)	F,T	171.80, 0.000
Dynamic	98.30 (34.73–268.66)	F,L,T	646.07 (408.83–790.35)	N,L,T,B	203.35 (88.94–546.95)	N,F	310.63 (130.80–723.90)	N,F,B	103.41 (33.61–228.45)	F,T	81.12, 0.000
Static	93.84 (26.37–213.30)	F,T	685.58 (375.59–910.24)	N,L,B	131.00 (50.00–308.78)	N,F,T	296.59 (162.84–672.35)	N,F,L,B	59.84 (20.94–160.83)	F,T	94.59, 0.000
ExplorDistance	10.34 (5.76–12.35)	T	11.29 (7.24–13.24)	T	10.57 (6.39–12.96)	T	12.79 (7.97–15.43)	N,F,L,B	10.51 (5.83–12.96)	T	46.40, 0.000
Dynamic	9.65 (5.76–11.95)	T	11.11 (7.24–13.53)	T	11.36 (6.32–13.45)	T	12.35 (6.97–15.56)	N,F,L,B	10.40 (4.94–12.88)	T	18.45, 0.000
Static	11.06 (5.82–12.41)	T	11.29 (7.31–13.08)	T	10.33 (6.39–12.05)	T	13.09 (8.76–15.09)	N,F,L,B	10.51 (7.48–13.28)	T	29.19, 0.000
Feature index	–0.40 (–0.66–0.049)	F,L,T	–0.14 (–0.40–0.12)	N,L,B	–0.32 (–0.50–0.061)	N,F,T	–0.12 (–0.40–0.20)	N,L,B	–0.39 (–0.57– 0.0058)	F,T	56.15, 0.000
Dynamic	–0.39 (–0.62–0.020)	F,T	–0.16 (–0.38–0.086)	N,B	–0.33 (–0.49–0.087)	-	–0.12 (–0.43–0.20)	N,B	–0.45 (–0.63– –0.055)	F,T	23.85, 0.000
Static	–0.40 (–0.72–0.077)	F,T	–0.10 (–0.42–0.20)	N,L,B	–0.30 (–0.54–0.054)	F,T	–0.10 (–0.36–0.20)	N,L,B	–0.35 (–0.54–0.0034)	F,T	33.69, 0.000

Table shows group medians and interquartile range (IQR) of all eight subjects per condition, results of Friedman’s ANOVA (last column) with test statistic value (χ^2^), degrees of freedom (df), number of values per condition included in the statistical analysis (*n*), and significance level (*p* value) and shows from which groups the respective group differed significantly (Diff, *p *<* *0.05) according to Dunn and Sidák’s *post hoc* multiple comparisons procedure (N = neutral, F = fear, L = lip smack, T = threat, B = blowing). Friedman’s ANOVAs did not include the blowing expressions to assure the same number of render types per expression category (no real blowing video). A second Friedman’s ANOVA was conducted leaving out the real videos (data not shown) and pairwise differences with the blowing expression are reported from this analysis. Effects were regarded as statistically significant at *p *<* *0.05; *p* values <0.001 were rounded to 0.000.

**Table 3:** Video type effect on all looking parameters investigated: cumulative fixation duration (FixDur), fixation number (FixNum), mean fixation duration (MeanFix), total looking time (LookT) on the entire face, the eyes and the mouth, as well as exploration distance (ExplorDistance) and the feature index

Lookingparameter	Dynamic	Static	χ^2^ (df = 1), *n* = 192, *p* value
	median (IQR)	median (IQR)	
FixDur face	1318.51 (1048.08–1512.81)	1269.04 (978.38–1497.71)	3.00, 0.083
FixNum face	4.87 (4.00–5.50)	4.82 (4.05–5.57)	0.89, 0.34
MeanFix face	273.05 (225.98–327.38)	263.35 (207.90–305.57)	21.33, 0.000
LookT face	1687.10 (1464.07–1862.00)	1655.48 (1400.45–1845.85)	2.08, 0.15
FixDur eyes	145.74 (42.89–265.92)	156.75 (57.86–326.73)	7.40, 0.0065
FixNum eyes	0.63 (0.21–1.25)	0.75 (0.22–1.47)	4.26, 0.039
MeanFix eyes	90.99 (32.88–157.91)	97.40 (43.25–163.75)	1.22, 0.27
LookT eyes	199.50 (72.57–363.35)	225.29 (94.66–443.80)	8.33, 0.0039
FixDur mouth	148.45 (37.80–322.05)	105.68 (32.49–306.04)	0.05, 0.82
FixNum mouth	0.43 (0.14–1.09)	0.41 (0.13–1.00)	0.60, 0.44
MeanFix mouth	99.79 (28.72–193.26)	75.88 (25.31–178.24)	6.08, 0.014
LookT mouth	216.93 (79.43–573.30)	204.05 (63.28–557.71)	2.08, 0.15
ExplorDistance	10.73 (6.23–13.56)	11.03 (7.07–13.49)	0.083, 0.77
Feature index	–0.26 (–0.50–0.063)	–0.23 (–0.49–0.097)	0.76, 0.38

Table shows group medians and interquartile range (IQR) of all eight subjects per condition and results of Friedman’s ANOVA (last column) with test statistic value (χ^2^), degrees of freedom (df), number of values per condition included in the statistical analysis (*n*), and significance level (*p* value). Groups were regarded as statistically significant at *p *<* *0.05; *p* values <0.001 were rounded to 0.000.

### Preferential looking (experiment 2)

**Figure 4.**
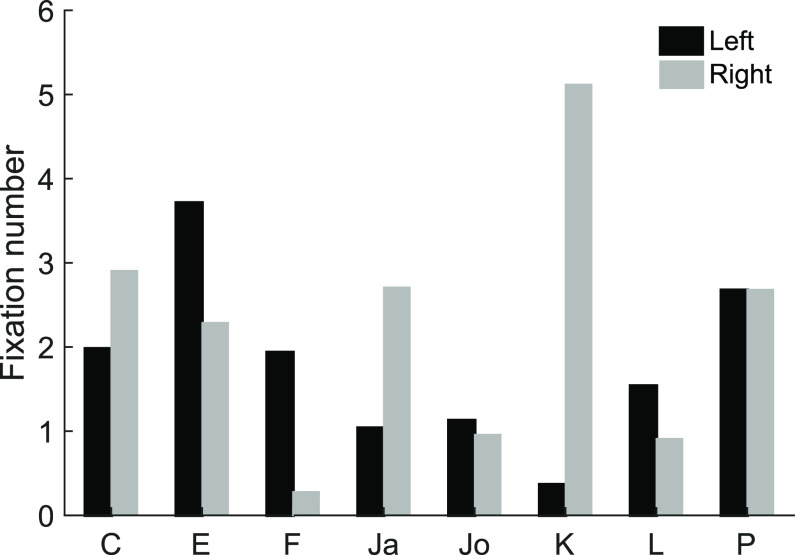
Monkeys’ average fixation numbers on each side during the preferential looking paradigm (experiment 2), revealing strong side biases of all monkeys except monkey P.

In experiment 2, instead of one central stimulus, two video clips were presented side by side. The two videos had the same expression category and video type (dynamic or static), but differed in render type. Analysis of this experiment quickly revealed that the monkeys’ looking behavior, confronted with a choice, was less driven by the stimuli, but rather by strong side biases: Friedman’s ANOVAs for the effect “side,” comparing how much the monkeys looked at the face on the left side versus the face on the right side, showed strong side biases in fixation number (χ^2^(1) = 37.70, *p *<* *0.001), cumulative fixation duration (χ^2^(1) = 36.78, *p *<* *0.001), total looking time (χ^2^(1) = 38.78, *p *<* *0.001), and mean fixation duration (χ^2^(1) = 27.38, *p *<* *0.001). Figure 4 shows that every monkey except one (monkey P) exhibited a clear bias toward one side of the screen. This very likely reflects prior overtraining on other tasks, as supported anecdotally after inquiring the monkeys’ training history, and/or idiosyncratic biases. Hence any further analysis of experiment 2 would not have been meaningful.

### Physiological measures

**Figure 5.**
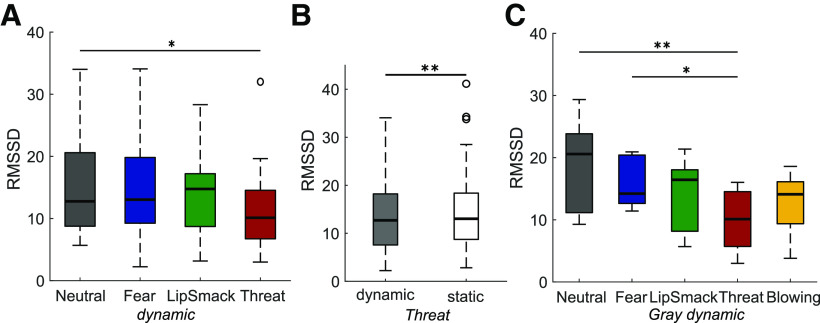
HRV, measured as RMSSD (*N* = 5). ***A***, All dynamic expressions compared, RMSSD was significantly lower in the threatening condition, indicating elevated arousal. ***B***, Dynamic threatening versus static threatening expressions. ***C***, Grayscale dynamic expressions only; *p <* *0.05, **p <* *0.01, ***p <* *0.001.

We recorded the monkeys’ electrocardiogram throughout experiment 1. The viewed dynamic expressions had a significant suppressive effect on the HRV, measured as the RMSSD (see Materials and Methods), as illustrated in Figure 5*A* (χ^2^(3) = 9.43, *p *=* *0.024). Although also static expressions tended to have a similar impact on the HRV, this effect did not reach significance (χ^2^(3) = 7.42, *p *=* *0.060). RMSSD was decreased in the dynamic threat condition compared with neutral (*p *=* *0.016). Similarly, when specifically looking at the video type effect within each expression group, the only significant effect was observed for the threatening expression, with a decreased RMSSD in the dynamic condition (χ^2^(1) = 9.00, *p *=* *0.0027; Fig. 5*B*). This indicates elevated arousal when viewing a moving threatening face. The effect of dynamic expressions was also investigated in each render type group separately, and it was revealed that the effect of the dynamic threatening expression was most strongly driven by the threatening grayscale avatar, which was the only render type group where the RMSSD in the threat condition was decreased significantly (χ^2^(4) = 15.20, *p *=* *0.0043) compared with neutral (*p *=* *0.0029) and compared with fear (*p *=* *0.041; Fig. 5*C*).

Pupil size analysis of our data did not yield any meaningful results, most probably because the signal was too corrupted due to tracking angle changes introduced by the exploratory gaze shifts. Undisturbed pupil size tracking requires steady fixation and carefully controlled luminance conditions, requirements that were precluded by our interest in unrestrained looking behavior.

### Reactive facial expressions

**Figure 6.**
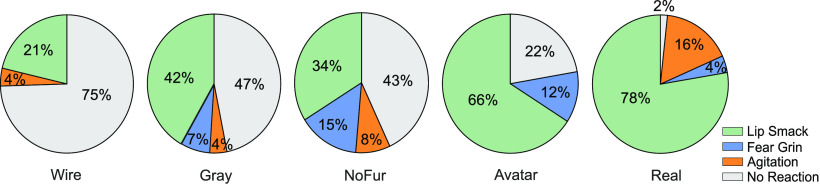
Average probability to show lip smacking, fear grinning, agitation (teeth grinding/tension yawning), or no reaction when viewing each render type. Monkeys (*N* = 3) were most likely to lip smack when seeing the real monkey and the naturalistic avatar (wireframe < real, *p *<* *0.001; furless < real, *p *=* *0.0018; wireframe < avatar, *p *=* *0.052). The probability for no reaction was highest for the unrealistic wireframe head (wireframe > gray, *p *=* *0.024; wireframe > furless, *p *=* *0.011; wireframe > avatar, *p *<* *0.001; wireframe > real, *p *<* *0.001; gray > real, *p *=* *0.017; furless > real, *p *=* *0.035). The highest probability to fear grin occurred toward the furless avatar (wireframe < furless, *p *<* *0.001, wireframe < avatar, *p *=* *0.041; real < furless *p *=* *0.041). Differences in agitated reactions were not significant.

Monkeys showed differential facial reactions on their initial encounter with the avatars. The render type significantly influenced the probability for lip smacking (χ^2^(4) = 23.44, *p *<* *0.001), fear grinning (χ^2^(4) = 18.87, *p *<* *0.001), and showing no reaction (χ^2^(4) = 41.91, *p *<* *0.001). Signs of agitation were only shown by monkey E and thus were not significantly different between the conditions. Monkeys lip smacked most toward the real video and the naturalistic avatar, whereas the furless avatar was the render type toward which they fear grinned the most, while seeing the wireframe avatar most frequently elicited no reaction (Fig. 6; Movie 1). The same effect was present when looking at dynamic and static videos separately. Neither the video type nor the expression alone significantly changed the probability for a certain facial reaction.

Movie 1. Monkey E reacting to grayscale, furless, real, and naturalistic render types; eye path overlaid on stimulus video (blue). 10.1523/ENEURO.0524-19.2020.video.1

## Discussion

The results show that the uncanny valley effect in monkeys can be overcome by using sufficiently naturalistic avatar stimuli. Consistently over all facial expressions, the monkeys avoided looking at the strange furless and gray avatar heads. The naturalistic avatar, the most unnaturalistic avatar, and the real monkey were looked at significantly more, whereby the difference between the real monkey and the naturalistic avatar is possibly due to familiarity of the observer monkeys with the depicted real monkey. This indicates an uncanny avoidance reaction for the less naturalistic but not the most naturalistic synthetic face, placing the naturalistic avatar on the other side of the uncanny valley. The monkeys’ facial expression reactions reflect this, as they tended to lip smack toward the real monkey and the naturalistic avatar. The furless avatar, on the other hand, was the most likely to elicit fear grinning and the very unnaturalistic wire head almost never gave rise to any kind of reaction. This supports the assumption that while the very unnatural wireframe avatar was not perceived as a monkey at all, both the real video and the naturalistic avatar were regarded as a conspecific warranting a positive approaching behavior, whereas the uncanny furless avatar elicited fear due to its eerie appearance. We illuminated that the avoidance of uncanny faces is associated with physiological arousal, as the clearest increase in arousal in terms of HRV was measured for moving grayscale threatening faces. This likely reflects negative emotions such as fear, similar to the uncanny aversion elicited in humans. Movement selectively amplified the avoidance of uncanny grayscale faces only, which had been predicted (Mori, 1970/2012), but so far not confirmed (Piwek et al., 2014). Indications for improper animation eliciting an aversion (Mori, 1970/2012; Tinwell et al., 2011; Piwek et al., 2014) were not obtained, as the uncanny valley emerged both for static and for dynamic faces.

Moreover, the visual exploration patterns of avatar faces were in accordance with reports in the literature on how monkeys look at photographs of conspecifics (Keating and Keating, 1982; Nahm et al., 1997; Guo et al., 2003; Gothard et al., 2004; Ghazanfar et al., 2006), with the avatars’ facial expressions modulating looking patterns in a way that the face parts characterizing the expression were mainly looked at, as it was the case for monkey face pictures (Gothard et al., 2004). Static expressions with the most salient features, i.e., the open mouth of the threat and the bared teeth of the fear displays, were looked at most. When the expression was dynamic, threatening faces caused a significant increase in arousal and were also scrutinized most but followed by socially highly relevant affiliative lip smacking and then submissive fear grin, whereas uninformative neutral and unnatural blowing faces received the least attention. The increased preference for dynamic lip smacking and the physiological response to dynamic threat, along with the small interest in blowing faces, indicates that the looking behavior toward moving faces was possibly more driven by the social meaning of the expressions, instead of salient features or movement alone. This conclusion is also supported by the fact that fixations on dynamic faces were longer, particularly on the areas exhibiting the strongest movements. The differential exploration of dynamic and static expressions underlines the importance of naturalistically animated avatar stimuli, which we implemented by resorting to motion capture technology.

However, the physiological results obtained from the heart rate data should be regarded cautiously, as the design of our study was suboptimal for the detection of significant heart rate reactions. The duration of one trial was only 3 s, making it difficult to detect changes in the oscillatory HR, which resides in a frequency range of 2–3 Hz at rest for rhesus monkeys. Because of the sluggishness of HR reactions, attempts to identify changes are usually based on recordings of 5 min and rarely down to 10 s in experimental settings, but not less (Shaffer and Ginsberg, 2017). A block design repeating the same expression would be conceivable, but this would entail the downside of habituation.

The reactive facial expressions of our monkeys represent an interesting proof of principle. It has been demonstrated before that macaque monkeys lip smack toward videos of conspecifics under experimental conditions (Mosher et al., 2011; Shepherd and Freiwald, 2018), and show contagious yawning (Paukner and Anderson, 2006). Social reactions toward computer animations have been recorded for chimpanzees (Campbell et al., 2009). However, to the best of our knowledge, our study is the first demonstration of non-ape primates reacting toward a virtual avatar. We can speculate why only three monkeys (C, E, and P) showed any reactions toward the videos. All these three were dominant monkeys and had never been exposed to face stimuli under experimental conditions before. Monkeys Ja, Jo, K, and L did not show any behavioral reaction toward any of the videos. Possible explanations for this could be the low rank in the dominance hierarchy in the case of monkey K, as dominant monkeys are more likely to lip smack video monkeys (Mosher et al., 2011) and initiate contact. Monkeys Ja, Jo, and L had a long history of participation in experiments involving images and videos of conspecifics, which is why their lack of reaction could reflect habituation. Monkey F, a rather young and submissive monkey who had not been involved in any experiment before, exhibited general signs of agitation like fidgeting in his chair and fear grinning toward all videos indiscriminately.

### Implications for the use of avatars in social cognition research

The absence of an uncanny valley effect for our naturalistic avatar, the affiliative behavioral responses elicited by this avatar, and the differential reactions toward the various dynamic facial expressions validate our avatar as a suitable stimulus for additional experiments involving social cognition in monkeys, providing us with a powerful tool to study social perception and social interactions in a standardized, dynamic, fully controllable setting.

The basic possibility of computer avatars to elicit an uncanny avoidance reaction in monkeys, shown by us and by Steckenfinger and Ghazanfar (2009), shows that it is crucial to validate an artificial social stimulus before use. Virtual avatars have been employed as stimuli in behavioral experiments with monkeys before (Paukner et al., 2014, 2018), and lately, neurophysiological investigations showed that face-selective neurons respond to monkey avatar faces and are modulated by changing the gaze direction or facial expression (Murphy and Leopold, 2019). However, the face avatar stimuli used were not tested for an uncanny valley response, at least not to our knowledge.

Wilson et al. (2019) recently developed a head avatar of a long-tailed macaque (*Macaca fascicularis*) and investigated the looking behavior of rhesus and long-tailed macaques toward it. The study failed to reveal any difference in viewing times between static images of real faces, naturalistic and unnaturalistic avatars. That was interpreted as support for the use of the avatar and against the presence of an uncanny aversion in macaque monkeys. This stands in contrast to Steckenfinger and Ghazanfar (2009) and to our study, which clearly show that an uncanny valley exists in monkeys. The lack of agreement could arise from the species incongruence of avatar and observers, the small number of subjects, the deviating experimental design and the confinement of the analysis to one dependent variable in the study of Wilson et al. (2019). In this experiment, only three rhesus monkeys were tested who were repeatedly presented with the same stimuli during a single session and only total (cumulative) fixation time on the faces was measured, which proved to be the least informative parameter in our study. Moreover, as the observer monkeys were rhesus macaques while the avatar stimulus was modelled after long-tailed macaques, the ethological validity of the synthetic stimulus is decreased, possibly introducing unpredictable unfamiliarity and irritation effects. The experiments on the 10 long-tailed macaques cannot be compared as the animals were free-ranging, could view each image for up to 60 s or change the image earlier by touching a target. As only static avatars were tested, the important role of facial movements (Mori, 1970/2012; Tinwell et al., 2011; Piwek et al., 2014; Chouinard-Thuly et al., 2017) was not addressed.

### Implications for the uncanny valley hypothesis

Our results corroborate the existence of an uncanny avoidance reaction in macaque monkeys, first shown by Steckenfinger and Ghazanfar (2009). Moreover, we confirm the second prediction of the original uncanny valley hypothesis that movement would deepen the uncanny avoidance. The emergence of the uncanniness reaction in our non-human primate relatives has ramifications for possible explanations of the phenomenon. Currently, several lines of explanation for the uncanny valley effect exist: one hypothesis assumes pathogen avoidance as the critical mechanism, proposing that facial aberrations are a sign of disease, triggering disgust as an evolved mechanism for avoiding a contagious disease. The more human-like and thus genetically related a character seems, the more sensitive we may be to such facial defects, as the perceived chance of contracting the disease in question increases (MacDorman and Ishiguro, 2006; MacDorman et al., 2009). This is only partly supported by Green et al. (2008), who observed higher interrater agreement on what ideal facial proportions are for more human-like faces. However, the tolerance regarding the acceptable range of facial features was not affected by human likeness. Other perceptual hypotheses suggest that uncanny faces remind humans of their own mortality, or that they fail to meet evolved aesthetic standards, shaped by the specialized face processing system. Yet other lines of explanation engage more cognitive mechanisms, including the violation of expectations by eliciting expectations for a human being, but failing to fulfill them, or category uncertainty about whether or not a given entity is human/real or not (for review, see Wang et al., 2015).

Our findings provide support for an evolutionary origin of the phenomenon, like threat avoidance driven by disgust or fear, or evolved aesthetic standards arising from the highly specialized face processing system. Brink et al. (2019) assumed that an evolutionary origin of the uncanny valley would require the phenomenon to be present already in very young children. As they failed to observe an uncanny valley reaction in children younger than nine years, exposed to human-like and machine-like robots, they discarded a phylogenetic basis. However, this argument is flawed. Although a functional trait appearing early during development is most probably innate, the reverse does not hold true. The ability to walk is evolutionary in origin and nonetheless not present in infants, and so are various cognitive capabilities. Although a preference for looking at faces (Johnson et al., 1991) and a proto-organization of face perception is present from birth (Deen et al., 2017; Livingstone et al., 2017), the face processing system undergoes perceptual learning (Nelson, 2001) and refinement of selectivity throughout childhood of humans (Behrmann et al., 2016) and monkeys (Livingstone et al., 2017). It is likely that these changes are also associated with a refinement of sensitivity for facial deviations through experience as indicated by Lewkowicz and Ghazanfar (2012), who found avoidance of an uncanny avatar by 12-month-old children, but not by six-month-olds. The findings of Brink et al. (2019), however, seem to be less related to refinement of the face processing system than to learning of cognitive associations regarding robots.

Explanations centering around category uncertainty whether or not the uncanny character is a real human/conspecific monkey, or around the violation of expectations on how a presumed fellow human or monkey is supposed to behave, seem unlikely according to our data, as the “valley” we found in our study was not located at the place on the realism-axis predicted by Mori. Not the most realistic artificial stimuli were subject to avoidance but those of intermediate realism. We show that monkeys evade uncanny faces, but reveal that high realism is not the factor evoking the uncanny quality in a synthetic face. Instead, abnormal features in the stimulus (in our case lack of fur with abnormal smoothness of skin and lack of natural coloring) seem to elicit the uncanny avoidance, whereas sufficiently naturalistic stimuli without eerie features are able to eliminate the avoidance. Several studies reporting an uncanny response of humans used stimuli with abnormal features, such as unnaturally large eyes, mismatched degree of realism of different face parts (Seyama and Nagayama, 2007; MacDorman et al., 2009; Lischetzke et al., 2017), or alteration by plastic surgery (Rosenthal-von der Pütten et al., 2019). Among the studies that failed to detect an uncanny valley were notably those deploying controlled, morphed stimulus sets varying only realism (MacDorman and Chattopadhyay, 2017; Kätsyri et al., 2019). As Seyama and Nagayama (2007) pointed out, some robots, dolls, or computer animations seem very pleasant although they are unrealistic, and conversely, humans differ in perceived levels of pleasantness although they are all real. Following Ockham’s razor, it is more parsimonious to assume that abnormal visual features in the stimulus, experienced as off-putting, elicit the avoidance reaction instead of invoking the elusive concept of realism. In the same vein, one might argue that also the uncanny response found in monkeys by Steckenfinger and Ghazanfar (2009) may have been a consequence of the lack of lifelike proportions, skin texture, and fur in the avatar termed realistic, whereas the unrealistic avatar was not perceived as a monkey at all, like the wireframe head in our study.

The uncanny valley literature is full of methodological shortcomings and conceptual fallacies (for review, see Wang et al. 2015), in part because the hypothesis was initially ill defined, with the hypothetical curve lacking a mathematical formulation as well as clearly defined dependent and independent variables, leaving researchers too many degrees of freedom. One could argue that what is currently investigated under the term uncanny valley is actually a collection of sometimes interacting psychological phenomena ranging from a simple fear of the unknown (Jentsch, 1997), in particular being startled by something believed to be animate actually being inanimate or vice versa, over evolutionarily developed threat avoidance (MacDorman et al., 2009) with an aversion toward facial aberrations (Seyama and Nagayama, 2007), to a fear of technology takeover. The latter is possibly inspired by science fiction media, as we tend to ascribe higher abilities to more naturalistic robots (Walters et al., 2008) and spontaneously apply social expectations to computers (Nass and Moon, 2000), but we cannot anticipate what robots are capable of, as they might not have human morals and at the same time superhuman capabilities. Even if robots looked exactly like humans, as long as we still know or, more accurately, believe that they are robots, we would probably experience an uncanny feeling, as happening in HBO’s *Westworld* [see also Giger et al. (2019) for an overview of the benefits and drawbacks of humanizing robots].

## Synthesis

Reviewing Editor: Gustavo Deco, Universitat Pompeu Fabra Decisions are customarily a result of the Reviewing Editor and the peer reviewers coming together and discussing their recommendations until a consensus is reached. When revisions are invited, a fact-based synthesis statement explaining their decision and outlining what is needed to prepare a revision will be listed below. The following reviewer(s) agreed to reveal their identity: Lars Rogenmoser.

The authors developed an improved naturalistic rhesus monkey face avatar overcoming the “uncanny” problem which may advance research in social cognition.

-The study design is poorly conveyed. Rationales for specific manipulations are not always provided (e.g., blowing expression) or in some cases as late as in the Result or Discussion section (e.g., deliberately “uncanny” manipulation in less realistic advatars).

-The results do not nessessarily support the authors’ conclusion given that their study design contains confounding factors. For example, their findings of “uncanny vally effect” and lack thereof may be confounded by realsim perception and responses to eerie stimuli. Furthermore, the experiment does not control for ethological importance.

-The dichotomy “naturalistic vs. realistic” needs an elaboration.

-The connections between aversion (e.g. uncanny) and avoidance behavior inclunding the physical responses and facial experessions are not self-evident. They need to be established, ideally in the introduction section.

Introduction

-References are missing in the first section.

-What does “CG” (line 69) stand for?

-The rationale behind artificial “blowing” expression needs explanation, especially since this stimulus condition has been dropped out of the analyses later on.

-The term “free viewing paradigm” needs an introduction/better explanation.

Materials and Methods

-How many monkeys were included during the development of the dynamic expression modeling? Details (e.g. number of trials) on the recording sessions are missing.

-Is the “real filmed monkey” one of the 8 subjects?

-Were the monkeys head-fixed during the actual experiment?

-Were the monkeys trained for gaze fixation? What liquid reward did they receive? Were they on water restriction?

-What constitutes a fixation? How were the paticular fixations quantified?

-I recommend mentioning the explanatory values of the two “measures of exploration”. In what way do they (differentially) contribute to the study?

-"(n = 6)” contradicts what is described in the text (line 213).

-Which “dependent variables"are the authors referring to (line 221). Please specifiy.

-It is not appropriate to use a parametrical post hoc procedure given their distributions of interest deviate from the normal distribution.

Results

-I recommend remaining descriptive in the Results section, relocating predictions/hyotheses or operationalizations to the Method section and any intepretations to the Discussion section.

-When working with non-parametric statistical tests, it is rather appropriate to report the median (instead of mean and SD).

Figure 1:

B: What are the units of the color scale?

The legend is hard to follow. Only one example is depicted whereas different combinations are mentioned. For what are the ROIs and how were they selected?

C: Needs a better description and explanation.

Figure 2,3,4,5,6,7

-I recommend mentioning the N in the legends since not all datasets are always included.

- Medians (perhaps including quartiles or boxplots) should be plotted instead of means since non-parametric analyzes were performed.

Tables

- The reported Ns are misleading. The sample size is supposed to be 8. Ultimately, the “group mean” is not clear and misleading.
