# Erratum: Siebert et al., “A Naturalistic Dynamic Monkey Head Avatar Elicits Species-Typical Reactions and Overcomes the Uncanny Valley”

**DOI:** 10.1523/ENEURO.0227-22.2022

**Published:** 2022-06-24

In the article, “A Naturalistic Dynamic Monkey Head Avatar Elicits Species-Typical Reactions and Overcomes the Uncanny Valley,” by Ramona Siebert, Nick Taubert, Silvia Spadacenta, Peter W. Dicke, Martin A. Giese, and Peter Thier, which was published online June 8, 2020, an incorrect formula was used to calculate the physiological measure “heart rate variability,” expressed as the root mean square of successive differences (RMSSD). As a result, several revisions should be made to the article. These errors do not affect the conclusion of the article. The online version has been corrected.

On page 6, the formula should read as:

$$\mathrm{RMSSD}=\surd (\frac{1}{n-1}({\left({\mathrm{RR}}_{1}-{\mathrm{RR}}_{2}\right)}^{\text{2}}+...+{\left({\mathrm{RR}}_{n-1}\;–\;{\mathrm{RR}}_{n}\right)}^{\text{2}})).$$

On page 12, in the “Physiologic measures” section, the first paragraph should read as:

“We recorded the monkeys’ electrocardiogram throughout experiment 1. When the viewed expression was threatening, it tended to have a suppressive effect on the HRV, measured as the RMSSD (see Materials and Methods), for dynamic expressions (χ^2^(3) = 6.94, *p* = 0.074; illustrated in Fig. 5*A*) and for static expressions (χ^2^(3) = 6.98, *p* = 0.072). When specifically looking at the video type effect within each expression group, the only significant effect was observed for the threatening expression, with a decreased RMSSD in the dynamic condition (χ^2^(1) = 4.84, *p* = 0.028; Fig. 5*B*). This indicates elevated arousal when viewing a moving threatening face. The effect of dynamic expressions was also investigated in each render type group separately, and it was revealed that the effect of the dynamic threatening expression was most strongly driven by the threatening grayscale avatar, which was the only render type group where the RMSSD in the threat condition was decreased significantly (χ^2^(4) = 14.72, *p* = 0.0053), depicted in Figure 5*C*.”

A corrected Figure 5 and its legend appear below.

**Figure 5.**
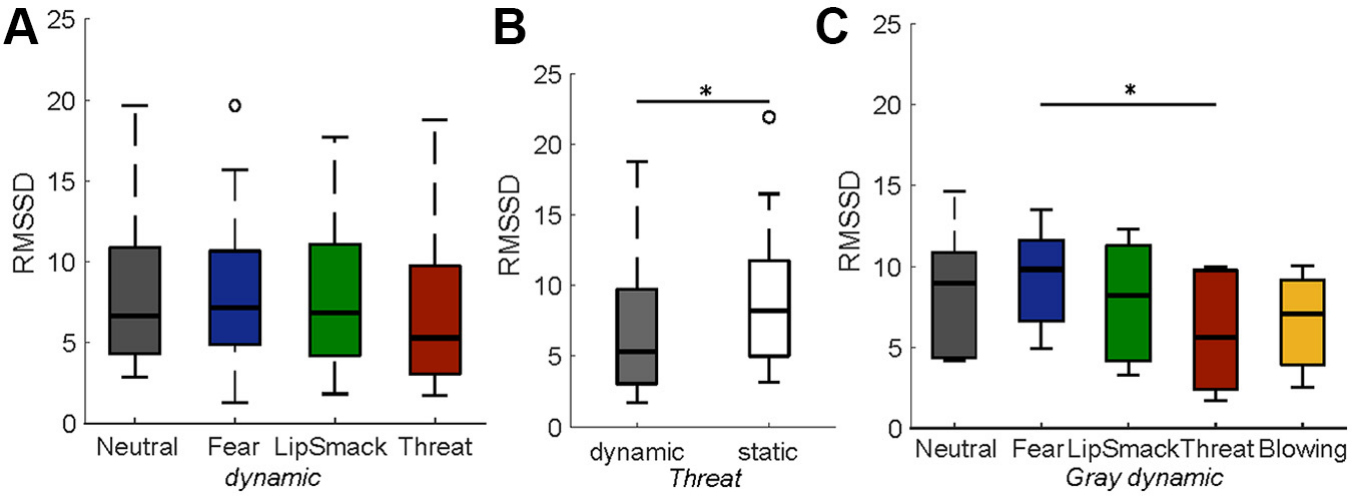
HRV, measured as RMSSD (*N *=* *5). ***A***, All dynamic expressions compared, RMSSD tended to be lower in the threatening condition, indicating elevated arousal. ***B***, Dynamic threatening versus static threatening expressions. ***C***, Grayscale dynamic expressions only; *p <* *0.05, **p <* *0.01, ***p <* *0.001.

